# The Composition of the Carcass, Physicochemical Properties, Texture and Microstructure of the Meat of D11 Dworka and P9 Pekin Ducks

**DOI:** 10.3390/ani12131714

**Published:** 2022-07-02

**Authors:** Dariusz Kokoszyński, Joanna Żochowska-Kujawska, Marek Kotowicz, Grzegorz Skoneczny, Svitlana Kostenko, Karol Włodarczyk, Kamil Stęczny, Mohamed Saleh, Marcin Wegner

**Affiliations:** 1 Department of Animal Breeding and Nutrition, Faculty of Animal Breeding and Biology, Bydgoszcz University of Science and Technology, 85084 Bydgoszcz, Poland; grzko000@utp.edu.pl (G.S.); karwlo19@gmail.com (K.W.); kamil.steczny@o2.pl (K.S.); marcin.wegner@op.pl (M.W.); 2 Department of Meat Science, Faculty of Food Sciences and Fisheries, West Pomeranian University of Technology, 71550 Szczecin, Poland; joanna.zochowska-kujawska@zut.edu.pl (J.Ż.-K.); marek.kotowicz@zut.edu.pl (M.K.); 3 Department of Animal Genetics, Breeding and Biotechnology, National University of Life and Environmental Sciences of Ukraine, 03041 Kyiv, Ukraine; svitlanakasijan@ukr.net; 4 Department of Poultry Production, Faculty of Agriculture, Sohag University, Sohag 82524, Egypt; bydg2016@gmail.com

**Keywords:** Pekin, Dworka, breed, meat quality, sex

## Abstract

**Simple Summary:**

The production and consumption of duck meat in the world have a centuries-old tradition. Currently, China is the largest producer of duck meat. In Europe, the top producers of duck meat are France, Hungary and Poland. The aim of this study was to compare Pekin ducks (conservative strain P9) and Dworka (breeding strain D11) in terms of weight and percentage of gutted carcass components, basic chemical composition, physicochemical properties of breast and leg meat as well as texture and microstructure of breast meat. Significant differentiation (*p* < 0.05) for the genotype was found in terms of carcass weight, the content of most chemical components, some physicochemical features of the pectoral muscles and legs, as well as the thickness of connective tissue, springiness and WB (Warner–Bratzler) shear force *m. pectoralis major.* These differences were mainly due to the different purposes of the ducks. Dworka ducks from the D11 strain are being improved in terms of their functional characteristics (breeding strain), while Pekin ducks from the P9 strain have been kept in Poland as a conservation herd (without breeding selection) for several decades.

**Abstract:**

The aim of the study was to determine the effects of genotype and sex on carcass composition and selected meat quality parameters of Dworka and Pekin ducks after two reproductive seasons. The research material consisted of 24 carcasses of Dworka ducks (breeding strain D11) and 24 carcasses of Pekin ducks from the herd of genetic resources (French Pekin, strain P-9). After cooling the carcasses (18 h, 2 °C), the pH values and electrical conductivity of the pectoral and leg muscles were determined, and then the carcass was dissected using a simplified method. After dissection, the pectoral and leg muscles were sampled for quality characteristics assessment. The Dworka ducks from breeding strain D11 compared to Pekin duck from conservative strain P9 were characterized by significantly (*p* < 0.05) higher carcass weight, protein and salt content, thermal drip, yellowness, thickness of *perymisium* and *endomysium* and lower water content of the pectoral muscle. Dworka ducks were also characterized by higher protein, salt and collagen content and higher electrical conductivity (EC_24_) of leg muscles than Pekin ducks. Regardless of genotype, male ducks had a higher gutted carcass weight with neck, salt content, muscle fiber cross-sectional area, fiber perimeter and diameters of pectoral muscle, and also higher protein and collagen, and lower fat content, pH_24_ and electrical conductivity of leg muscles. The genotype by sex interaction was significant (*p* < 0.05) for water, protein, fat content, perimisium thickness, cohesiveness, springiness of the pectoral major muscle, and for water content and protein of the leg muscles. The results obtained in this study demonstrate the effects of genotype and sex on the nutritional value and some technological parameters of duck meat. The studied ducks after two reproductive seasons satisfactorily meet the requirements of duck meat for consumers and duck meat processing plants.

## 1. Introduction

In Poland, hybrids from parents imported from the French company Grimaud Frères Sélection (Star 53 HY hybrid) or Orvia (ST5 Heavy hybrid) and parents from the English company Cherry Valley Farms Ltd. (SM3 Heavy hybrid) are used for the intensive production of Pekin ducks. Hybrids AF51 or FA15 after parental ducks distributed by Poultry Breeding Farm in Tuchola (Poland) are used to a lesser extent. The breeds/families of ducks from genetic resource herds and Dworka ducks [1,2] are particularly suitable for the extensive (farmyard) system of keeping ducks.

The domestic commercial material of ducks (broilers) is characterized by good utility value, and the performance results of duck broiler flocks, determined with the European Productivity Index (EPI), are considered good. Nevertheless, both the purchase price of live animals and the interest of consumers in purchasing carcasses and duck meat are still relatively low in Poland. Duck meat is still perceived as a noble and more expensive assortment used on special occasions [2].

Native breeds of ducks are valuable materials for scientific research in many areas. The slaughter value and quality of duck meat from genetic resource herds has been the subject of numerous research and scientific studies, but the obtained knowledge is still incomplete [3,4,5,6,7,8,9]. In recent years, livestock, including ducks, have also begun to be kept in agritourism farms where animal populations are not too large. These farms try to offer their customers high quality dishes, which is greatly influenced by the quality of the raw material. Research aimed at determining the quality of duck carcass and meat is of great importance for producers and consumers of duck meat [10].

In this study, the carcass composition and meat quality of Dworka ducks from breeding strain D11 and Pekin ducks from conservative strain P9 were compared. Dworka ducks were first bred in Poland at the end of the 1990s as a result of crossing black-colored Cayuga ducks with white-colored Pekin ducks. Dworka ducks have black and white (variegated) plumage, a black or flesh-colored beak and flesh-colored or light yellow feet. At 7 weeks of age, they weigh approx. 3000 g. In a period of six months, one duck lays an average of 140–150 eggs in the first year of reproduction. They are characterized by high resistance to unfavorable environmental conditions, calm temperament and good foraging, including good use of farm fodder. Ducks of this breed are distinguished by a fairly good level of meat and reproductive features. They are characterized by a well-muscled and long brisket [11,12]. French Pekin ducks (P9 conservative strain) were bred from chicks hatched from eggs purchased from the French company Jansen in 1978. In 1983, these ducks were considered as a conservative strain and were subject to protection of the quality of genetic resources. The P9 duck has white plumage, and its beak and paws are yellow-orange. When they are 7 weeks old, the drake weighs an average of 2400–2450 g, while the duck weighs about 2300 g. Adult birds weigh on average from 2850 to 3100 g. During a six-month period, one duck lays about 157 eggs in the first year of reproduction and about 127 eggs in the second year [12].

In recent years, the results of research on the influence of genotype and sex on the carcass composition and meat quality of ducks have been presented in several scientific articles [13,14,15,16,17,18,19,20,21], but the obtained knowledge is still incomplete and further research on this subject is needed. So far, only few scientific studies [16,22,23,24,25] have presented results concerning the carcass composition and quality characteristics of duck meat after reproductive use.

The available literature lacks information on the carcass characteristics and meat quality of Dworka ducks after reproductive use. There are also no comparative studies on the carcass composition and meat quality of Pekin P9 and Dworka D11 ducks after two reproductive seasons, which encouraged the execution of this study.

The aim of the study was to determine the effects of genotype and sex on carcass composition and selected meat quality parameters of D11 Dworka and P9 Pekin ducks after two reproductive seasons.

## 2. Materials and Methods

### 2.1. Carcass Collection and Evaluation of Carcass Traits

The research material consisted of 24 carcasses of Dworka ducks (breeding strain D11) and 24 carcasses of Pekin ducks from the genetic resources herd (French Pekin, conservative strain P9), with 12 carcasses of males and 12 carcasses of females from each strain. The carcasses were obtained after the slaughter of the ducks after two reproductive seasons (at the age of 113 weeks). According to the information of the breeder, during the reproductive season, the ducks from both assessed strains were kept in the same building with regulated environmental parameters and were fed the same diet (complete compound feed from the feed factory) with the same nutritional value. A commercial complete feed mixture for parent ducks was ground and contained 16.13% CP, 11.09 MJ metabolizable energy, 5.10% crude fat, 4.03% crude fiber, 11.38% crude ash, 3.01% calcium, 0.50% phosphorus, 0.17% sodium, 0.37% methionine and 0.82% lysine. Components of the feed mixture were wheat, corn, soybean meal from hulled GMO soybeans, triticale, barley, oats, calcium carbonate, sunflower seed extraction meal, soybean oil, fish meal, monocalcium phosphate, sodium chloride and sodium bicarbonate.

The time of slaughter was related to the liquidation of duck flocks in accordance with the breeding program. Birds originating from the Waterfowl Genetic Resources Station in Dworzyska near Poznań (Poland) were slaughtered in a poultry processing plant, according to applicable regulations in the EU and the Polish poultry industry. The research was carried out with the consent of the Local Ethical Committee for Experiments with Animals in Bydgoszcz, Poland (Resolution No. 17/2010 of 23 June 2010).

After transporting them to the laboratory, the purchased gutted carcasses with their necks, without giblets, were cooled in a refrigerator for 18 h at a temperature of 2 °C. After cooling down, the weight of each carcass was determined individually with the WLC 6/12/F1/R electronic scale with an accuracy of 0.1 g. After weighing the carcasses, the acidity and electrical conductivity of the pectoral and leg muscles were determined. Then, the dissection of the carcasses began. Each carcass was dissected using a method developed by Ziołecki and Doruchowski [26]. Wings with skin, neck without skin, skin with subcutaneous fat from the whole carcass without skin from the wings, saddle fat, leg muscles (all thigh muscles and drumstick on both legs), pectoral muscles (fillet and tenderloin on both breast) and carcass remains were separated from the individual carcasses. The elements extracted during the dissection were weighed on the above-mentioned electronic scales. Based on the data on the weight of the gutted carcass with the neck and its parts, the percentage share of the individual parts in the carcass was calculated. After dissection of the duck carcasses, samples were taken from the leg muscles and the pectoral muscles to determine the chemical composition (water, protein, intramuscular fat, collagen and salt content), thermal drip, meat color and samples of the superficial pectoral muscle for the determination of texture parameters and microstructure features.

### 2.2. Basic Chemical Composition

The basic chemical composition, i.e., content of water, protein, intramuscular fat, collagen and salt, was determined by near-infrared transmission spectrometry (NIR) using the FoodScan (FoodScan Laboratory, Foss, Cheshire, UK) apparatus. The spectral range of reflectance used in NIR was 850–1050 nm. For this purpose, 90 g samples of the breast or leg meat were taken from the carcass, and then minced using an electric meat grinder (2 mm diameter sieve) produced by Zelmer (Rzeszów, Poland).

### 2.3. Physicochemical Characteristics

Before the dissection, the acidity of both the greater pectoral muscles and the shank muscles were measured 24 h after slaughter. Meat acidity measurements were performed with an accuracy of 0.01 using a pH-Star CPU pH meter equipped with a glass electrode for measuring pH of meat. Twenty-four hours after slaughter, the electrical conductivity (mS/cm) of the same muscles was also measured with the LF-Star CPU device. During the measurement, the electrodes of the conductometer were placed along the muscle fibers at an angle of 90°. The measurement was performed with an accuracy of 0.1 mS/cm.

Determination of thermal drip of meat from breasts and legs was performed using the Walczak method [27]. Meat samples were placed for 10 min in a water bath with a water temperature of 85 °C. The samples weighing 20 g each were previously ground and wrapped in hygroscopic gauze. After taking the samples out of the water bath, the meat was cooled at 2 °C for 30 min. After this time, they were weighed a second time using the electronic scale WLC6/12/F1/R (Radwag, Radom, Poland). Based on the difference in weight of the sample before and after heat treatment, the weight loss of the sample was calculated as a percentage.

After carcass dissection, the meat color parameters were determined on the inner surface of the raw muscles of the legs and pectoral muscles. The measurement was performed in accordance with the CIELab system. The following color parameters were determined: color brightness (L*), red color intensity (a*), yellow color intensity (b*). Measurements were made using a Konica Minolta CR410 (Konica Minolta, Tokyo, Japan) colorimeter. The measurement area was 50 mm in diameter. The calibration of the meter was performed using a standard CR410 white plate, taking into account the data needed for the calibration of the device (Y = 94.40, x = 0.3159, y = 0.3325).

### 2.4. Texture Measurement

The texture of *m. pectoralis major* was tested on a Stable Micro Systems TA.XT plus apparatus (Stable Micro Systems, United Kingdom) using the Texture Profile Analysis (TPA) test and the Warner–Bratzler (WB) test. The tests were carried out in accordance with the texture profile analysis procedures [28]. Muscles were placed separately in plastic bags, cooked in a water bath at 72 °C until the geometric center reached 70.2 °C, cooled to a temperature of approx. 12 °C and then stored at 4 °C for 12 h for texture measurements. In the TPA test, a 0.62 cm diameter shaft parallel to the sample muscle fiber decreased to 80% of its original height (16 mm). A traverse speed of 50 mm/min and a load cell of 50 N were used. The force–deformation curve obtained in the TPA test was used to calculate the hardness, cohesiveness, springiness and chewiness of the meat. The maximum cutting force necessary to cut the sample were determined in the Warner Bratzler (WB) test. The working speed of the traverse was assumed to be 50 mm/min; samples with a cross-section of 10 × 10 mm and height of 20 mm were cut parallel to the muscle fibers using a triangle knife. The TPA test and the WB test were repeated 5 times for each sample.

### 2.5. Measurements of Structure Elements

In order to perform histological examinations, samples of *m. pectoralis major* (pectoral muscle) were collected. From each sample, three sections were taken parallel to the path of the muscle fibers. The sections measuring 0.5 × 0.5 × 1 cm were taken from the center of the pectoral muscle. At a later stage, the samples were fixed with Sannomiya fluid, dehydrated with alcohol and benzene, and embedded in paraffin in the form of blocks. Blocks were cut with a microtome and 10 µm sections were placed on slides and counterstained with hematoxylin and eosin [29]. Then, the trials were closed in Canadian balm. Computer image analysis MultiScanBase version 13 was used to measure the microstructure properties of *m. pectoralis major*. The following features of the microstructure of the pectoralis major muscle were determined: the cross-sectional area, the circumference of the fiber, the vertical diameter of the fiber (V), the horizontal diameter of the fiber (H) and the thickness of the perimysium and endomysium. A total of 144 preparations of *m. pectoralis major* were used to determine the microstructure. About 200 muscle fibers in total were measured on each slide, and 150–200 measurements of the thickness of the connective tissue (perimysium and endomysium) were taken. A magnification of 100× was used. The shape of the muscle fibers was calculated as the ratio of the vertical diameter (V) to the horizontal diameter (H). The closer this value was to 1, the more regularly shaped the fiber was.

### 2.6. Statistical Analysis

The data collected during the research on the composition of the carcass and meat quality of ducks were statistically characterized. At the beginning of the statistical analysis, the Shapiro–Wilk test was applied to verify the normal distribution of the test carcass characteristics and meat quality of the ducks. For each tested feature, the arithmetic mean value (x) and standard deviation (sd) were calculated. The influence of genotype and sex on the examined traits of ducks was investigated using a two-way analysis of variance. Finally, the following linear model was used: *Y_ijk_* = *μ* + *a_i_* + *b_j_* + (*a·b*)*_ij_* + *e_ijk_*, where *Y_ijk_* is the value of the analyzed trait, *μ* is the overall mean for the tested trait, *a_i_* is the effect of i-th genotype, *b_j_* is the effect of j-th sex, (*a·b*)*_ij_* is the genotype by sex interaction, *e_ijk_* is the random error.

The calculations were made using the SAS computer program, version 9.4. [30]. Tukey’s test was used to verify significant differences (at level *p* < 0.05) between strains as well as males and females.

## 3. Results

### 3.1. Carcass Weight and Composition

The compared duck strains differed (*p* < 0.05) in terms of carcass weight. At 113 weeks of age, significantly heavier carcasses were obtained from Dworka ducks (breeding strain D11) compared to Pekin ducks (conservative strain P9). Regardless of their origin, males had a significantly greater weight of the gutted carcass with the neck (without the edible offal) than females. The genotype, sex of ducks and genotype–sex interactions were not statistically significant for the percentage of carcass components of the studied Dworka (breeding strain D11) and Pekin (conservative strain P9) ducks. Dworka ducks had higher percentages of breast muscle (*p* = 0.519), leg muscles *(p* = 0.660), abdominal fat (*p* = 0.665) and carcass remainders (*p* = 0.537) and lower percentages of skin with subcutaneous fat (*p* = 0.511), neck (*p* = 0.476) and wings (*p* = 0.164) compared to conservative P9 Pekin ducks (Table 1).

### 3.2. The Chemical Composition of Meat

Analyzing the data summarized in Table 2, it can be concluded that the breast muscles of Dworka ducks (breeding strain D11) contained significantly less water and more protein and salt, compared to the breast muscles of Pekin (conservative strain P9) ducks. Dworka ducks had significantly higher protein, collagen and salt content in the leg muscles compared to the Pekin ducks. Regardless of their genotype, males were characterized by higher salt content in the pectoral muscles and higher protein, collagen and salt content in the leg muscles, as well as lower fat content in the leg muscles than in females. The genotype–sex interaction was statistically significant for the water, protein and fat content in the pectoral muscles, and for the water and protein content in the leg muscles.

### 3.3. Physicochemical Characteristics of Meat

We also determined the effects of genotype and sex on the physicochemical characteristics (pH_24_, EC_24_, thermal drip, color changes L*, a*, b*) of the pectoral muscles and the muscles of the legs. The genotype of birds had a significant effect on the thermal drip and the intensity of the yellow color of the pectoral muscles, as well as the electrical conductivity of the leg muscles (Table 3 and Table 4). Dworka ducks from breeding strain D11 were characterized by significantly greater thermal drip and yellow color intensity of the pectoral muscles, as well as significantly greater electrical conductivity of the leg muscles compared to the Pekin ducks from conservative strain P9. The sex of the birds had no significant effect on the assessed physicochemical properties, except for the pH_24_ and electrical conductivity of the leg muscles. Significantly higher pH_24_ and electrical conductivity of the leg muscles were found in females compared to males. The genotype–sex interaction for the examined physicochemical traits was not confirmed statistically.

**Figure 1:**
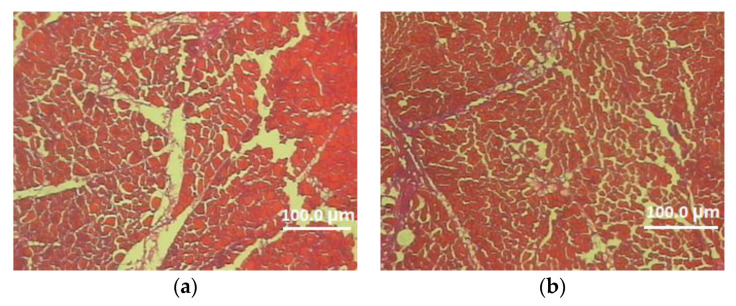
Cross-section of the superficial pectoral muscle of male (**a**) and female (**b**) 113-week-old Pekin P-9 strain (magnification 100×).

**Figure 2:**
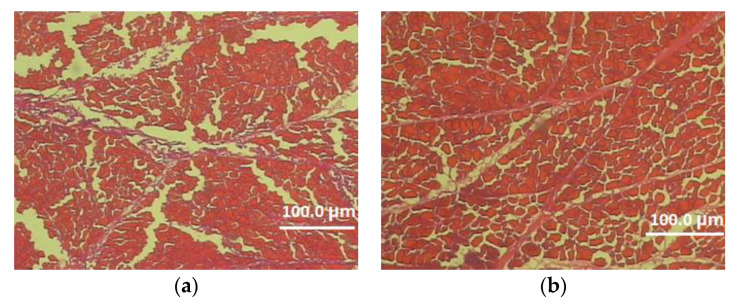
Cross-section of the superficial pectoral muscle of male (**a**) and female (**b**) 113-week-old Dworka D11 strain (magnification 100×).

In this experiment, the effects of genotype and sex on the microstructure characteristics of the pectoralis major muscle were also determined (Table 5, Figure 1 and Figure 2). Analysis of the data included in Table 5 demonstrated a significant effect of the genotype on the thickness of the perimysium and endomysium. After two reproductive seasons, at the age of 113 weeks, Dworka ducks from breeding strain D11 had a significantly greater thickness of connective tissue (perimysium and endomysium) than Pekin ducks from conservative strain P9. The sex of the birds had a significant effect on the muscle fiber cross-sectional area, circumference, horizontal and vertical diameters of the muscle fiber as well as the thickness of the endomysium. Significantly higher values of the above-mentioned structure elements were recorded for the D11 Dworka ducks than for P9 Pekin. The genotype–sex interaction was significant for perimysium thickness.

The compared breeds of ducks (Dworka vs. Pekin) differed significantly (*p* < 0.05) in springiness and WB shear force of heat-treated breast muscle (including *pectoralis major*). P9 males were characterized by significantly higher muscle springiness compared to P9 females and Dworka males and females (strain D11) (Table 6). Moreover, higher tenderness (significantly lower WB shear force values) was found in pectoralis major of 113-week-old Dworka females compared to Dworka males, as well as male and female Pekin ducks. The genotype–sex interaction was significant for the cohesiveness and springiness of breast muscle of the compared breeds of ducks.

## 4. Discussion

The obtained results indicate that the genotype of ducks has a significant effect on their carcass weight and on most of the determined chemical components, some physicochemical parameters of the meat as well as the thickness of the perimysium and endomysium. The genotype of ducks had no significant effect on the composition of the carcass and most of the microstructure features of pectoralis major. Significant differences in the carcass weight of P9 conservative Pekin strain and D11 Dworka breeding strain ducks were probably related to the breeding selection carried out in Dworka ducks for high body weight in the initial period of rearing, i.e., at the age of 3 and 7 weeks. P9 Pekin ducks, on the other hand, are kept as genetic resources, so they are not improved in terms of functional characteristics, including body weight. In the P9 strain, duck are only removed from the herd due to health condition and abnormal body structure. The studies by Kokoszyński et al. [23] found a lower weight of male carcasses (1826 g) and a greater weight of female carcasses (1927 g) obtained from 110-week-old P9 ducks compared to the assessed ducks of the same strain. On the other hand, Lewko and Gornowicz [31] reported a lower carcass weight of 10-week-old drakes (2059 g) and ducks (1769 g) from the D11 strain, which may indicate a further increase in carcass weight (and BW) after 10 weeks of duck rearing. The same authors [31] also found a significant increase in the carcass weight of D11 males (increase by 177 g) and a slight increase in CW of females (by 45 g) between 8 and 10 weeks of age. This may indicate a much faster growth rate of D11 males than females of this herd in this rearing period. In these studies, no significant differences were found in terms of carcass composition. Considering the scientific literature, Kokoszyński et al. [9] demonstrate significant differences in the percentage of pectoral muscles and fat in carcasses of 8-week-old Pekin ducks from genetic resources of (conservative herds) P33 (Polish Pekin), P8 (Danish Pekin) and P9 (French Pekin). Pekin ducks from P33 (Polish Pekin), P8 (Danish Pekin) and LsA (English Pekin) herds at 110 weeks of age did not differ in the percentage of carcass components [23], which may indicate the disappearance of diversification among the studied duck flocks, and is consistent with the results of our research. In turn, Kokoszyński et al. [23] observed a higher proportion of breast muscle and leg muscle, and a lower proportion of abdominal fat in the carcasses of 110-week-old P9 ducks compared to 113-week-old P9 ducks. In turn, Lewczuk and Gornowicz [31] reported a higher percentage of pectoral muscles and leg muscles in the carcass of 10-week-old P9 ducks (in total, 30.44% males, 29.26% females) compared to the results of these studies (27.8% in P9 males, and 26.9% in females in relation to the weight of the gutted carcass with the neck). The content of skin with subcutaneous fat in carcasses of 113-week-old P9 and D11 ducks did not exceed 24% and was lower than in carcasses of 112-week-old P33 (Polish Pekin) and P8 (Danish Pekin) ducks, and higher than in carcasses of LsA (English Pekin) ducks [9].

The studied Dworka and Pekin ducks were also compared in terms of their basic chemical composition, i.e., content of water, protein, intramuscular fat, collagen and salt in the breasts and leg muscles. In the studies by Bernacki et al. [32], higher water content and lower protein content were found in the pectoral muscles of 8-week-old Pekin (Star 63, AP57, PP5) and Dworka (CaA15) hybrid ducks compared to the assessed P9 and D11 ducks. Kokoszyński et al. [9] found lower content of water, protein and collagen in the pectoral muscles of 112-week-old P33 (Polish Pekin), P9 (French Pekin) and LsA (English Pekin) ducks compared to 113-week-old P9 and D11 ducks. P33, P9 and LsA ducks at 112 weeks of age had lower water content but higher protein and collagen content in the leg muscles than the assessed P9 and D11 ducks. Another experiment [23] found that 110-week-old P9 (French Pekin), K2 (miniature duck) and KhO1 (hybrid of Khaki Campbell and Orpington duck) ducks differ significantly in terms of water and fat content in the breast muscles as well as water, protein and fat in the leg muscles. In our study, we found high protein content of breast and leg muscles. Breast muscles contained more protein than leg muscles. Kokoszyński et al. [18] found higher protein and fat content in breast muscles of 49-day-old commercial hybrid Pekin ducks (Star 53 HY hybrid, 23.2% males, 23.4% females) compared to Polish native Pekin ducks (strain P33, 21.7% males, 21.4% females), which is consistent with the results of this study. Higher protein and fat contents in breast muscles were found in selected D11 ducks than in non-selected P9 ducks. In turn, Muhlisin et al. [33] observed higher protein content (21.84%) in breast muscles of Korean native ducks compared to imported commercial Pekin ducks (20.19%). The differences in the content of protein, fat and collagen obtained in these studies may have an impact on the nutritional and technological value of the meat of the compared breeds of ducks. Significant interactions of genotype and sex for the protein content of the meat from the breast and legs of the tested ducks may indicate a different degree of conversion of the protein contained in the feed mixture into the protein of male and female Pekin ducks from conservative strain P9 and ducks of Dworka from breeding strain D11.

Power of hydrogen (pH) value is a good indicator of the quality of meat. Bernacki et al. [32] found higher acidity (lower pH_24_ values) of the breast and leg muscles of 8-week-old broiler ducks of the Pekin type (AP57, Star 63, PP54 hybrid) and Dworka type (CaA15 hybrid) compared to the acidity of the P9 and D11 leg muscles after reproductive use. In turn, Kokoszyński et al. [9] found a lower pH_24_ as well as higher electrical conductivity (EC_24_) and thermal drip of the pectoral and leg muscles of 112-week-old ducks P33 (Polish Pekin), P8 (Danish Pekin) and LsA (English Pekin) compared to the results of this study carried out on 113-week-old ducks of the P9 and D11 strains. The high pH_24_ values of the pectoral and leg muscles of 113-week-old Dworka (breeding strain D11) and Pekin (conservative strain P9) ducks are probably related to the fatigue (exhaustion) of old ducks, and thus the lower content of glycogen before slaughter, which resulted in less acidification of the meat (higher pH_24_) compared to young slaughter birds.

Color is another parameter of meat quality that is important to consumers and the processing industry. In this study, no significant effect of genotype on the lightness and redness of the pectoral and leg muscles and yellowness of the leg muscles was found. On the other hand, in the studies by Kokoszyński et al. [23], lightness (L*), redness (a*) and yellowness (b*) of the pectoral muscles of 110-week-old P9 ducks were higher those of the tested P9 ducks at 113 weeks of age. In the same study [23], significantly higher L*, slightly higher a* and lower b* values were found, which may indicate a darker color of the leg meat of the studied P9 and D11 ducks. Heo et al. [14] stated in their study that commercial hybrids and Korean native ducks did not differ significantly in the L*, a* and b* values of breast muscles. In turn, Kwon et al. [34] reported significantly darker color of breast muscles from 56-day-old Korean native ducks compared to imported commercial duck hybrids aged 42 days. As in the study of Gornowicz et al. [35], sex had no effect on the L*, a* and b* values of breast and leg muscles from the tested ducks. However, contrary results were reported in the study of Michalczuk et al. [36], where breast muscles from 7-week-old female P-44 ducks were characterized by significantly higher redness (a*) compared to male muscles. According to Fletcher [37], meat color depends mainly on the myoglobin content, chemical structure of heme and pH of the meat. Myoglobin content is determined mostly by bird species, age and muscle type.

There was no significant effect of the genotype on the microstructure of pectoralis major muscle in D11 and P9 ducks, except for the thickness of the perimysium and endomysium. In turn, Kokoszyński et al. [16] demonstrated significant differentiation in terms of fiber cross-sectional area, fiber perimeter and fiber vertical diameter of pectoralis major muscle of P9 (French Pekin), KhO1 (hybrid formed with the Khaki Campbell and Orpingthon Fauve ducks) and K2 (mini duck, crossbreed with Pekin and wild mallards *Anas platyrchynchos* ducks) ducks at 110 weeks of age. On the other hand, Huo et al. [20] inform about significant differences in terms of fiber cross-sectional area, fiber diameters and fiber density between fast-growing Cherry Valley duck and slow-growing Small-sized Pekin and Liancheng White ducks. In turn, Balowski et al. [38] reported lower perimysium thickness (15.9 µm) and higher endomysium thickness (3.1 µm) of pectoralis major muscles of mallards ducks over 12 months old compared to the studied Dworka (breeding strain D11) and Pekin (conservative strain P9) ducks at 113 weeks of age.

It was the first time in the authors’ research that the texture parameters (hardness, cohesiveness, springiness, chewiness, gumminess, WB shear force) of the pectoralis major muscle of Dworka ducks after reproductive use were determined. Heat-treated breast muscles of 110-week-old P9 [23] (Pekin of French origin), K2 (mini duck, cross of wild mallard and Pekin duck) and KhO1 (crosses of Khaki Campbell males and Orpington Fauve females duck) ducks were found to be characterized by lower springiness and chewiness compared to the pectoral muscles of 113-week-old P9 (Pekin ducks) and D11 (Dworka Ducks) ducks. In the same experiment [23], higher hardness and gumminess were recorded in the pectoralis major muscle of K2 ducks, and lower in the muscles of P9 and KhO1 ducks than in the tested P9 and D11 ducks. The pectoral muscles of Pekin (P9) and Dworka (D11) ducks analyzed in this study were characterized by higher tenderness (lower WB shear force values) compared to P9 ducks, and they were less tender than 110-week-old KhO1 ducks described by Kokoszyński et al. [23]. In turn, Balowski et al. [38] reported similar hardness and cohesiveness, and lower springiness and chewiness of the pectoralis major muscle of male mallard wild ducks (*Anas platyrchynchos* L.) aged over 12 months compared to the pectoral muscles of Pekin (strain P9) and Dworka (strain D11) ducks at 113 weeks of age. Significantly lower WB shear force values of Dworka females indicate significantly higher tenderness of the pectoralis major muscles of these birds. Higher tenderness of the greater pectoral muscle of D11 females was associated with lower hardness. Significant interactions of genotype and sex in terms of the thickness of the perymisium *m. Pectoralis major* were associated with the diversified reaction of males and females in a given strain, which may indicate the specificity of the studied strains. Janicki and Buzała [39] report that the tenderness of meat depends on the content, composition and structure of the intramuscular connective tissue (epimysium, perimysium, and endomysium), as well as the degree of post-slaughter muscle fiber protein degradation.

## 5. Conclusions

In summary, after two reproductive seasons, the genotype of ducks had a significant effect on carcass weight, water, protein, salt content, thermal drip, yellowness, thickness of connective tissue (*perymisium, endomysium*), springiness and WB shear force of pectoral muscles, as well as protein, collagen, salt content and electrical conductivity (EC_24_) of the leg muscles. The sex of the birds had a significant effect (*p* < 0.05) on carcass weight, salt content, fiber cross-section area, fiber perimeter, fiber horizontal and vertical diameters and *endomysium* thickness.

The obtained results indicate that the carcasses and meat of the studied duck strains after two reproductive seasons may be useful for consumers and meat processing plants due to the favorable tissue composition of the carcasses, high nutritional value and good technological qualities.

## Figures and Tables

**Table 1:** Mean values (×) and standard deviation (sd) of the percentage of elements in the carcasses of 113-week-old ducks.

Trait		Genotype (G)–Sex (S)				*p*-Values		
		Dworka (Strain D11)		Pekin (Strain P9)		G	S	G × S
		Male	Female	Male	Female			
carcass weight (g)	× sd	2167.2 ^a^ 179.8	2058.0 ^a^* 216.0	1937.0 ^a^ 184.0	1724.6 ^b^* 181.1	0.001	0.005	0.354
breast muscle (%)	× sd	16.8 4.4	15.6 2.3	16.4 1.4	15.2 2.4	0.519	0.053	0.954
leg muscle (%)	× sd	11.0 1.8	11.4 1.6	10.5 2.0	11.5 2.1	0.660	0.237	0.618
skin with fat (%)	× sd	22.3 3.9	22.6 4.0	23.5 3.5	23.0 4.4	0.511	0.971	0.736
abdominal fat (%)	× sd	0.7 0.3	0.5 0.3	0.5 0.3	0.6 0.3	0.665	0.863	0.374
neck (%)	× sd	7.5 0.9	7.4 1.1	7.8 1.0	7.5 1.0	0.476	0.439	0.713
wings (%)	× sd	13.1 1.1	13.6 1.1	14.0 1.1	13.6 1.2	0.164	0.876	0.185
remainders (%)	× sd	28.8 4.4	28.7 2.3	27.3 4.0	29.0 3.2	0.537	0.440	0.384

^a, b^*p* < 0.05, means with different letters are statistically different. * *p* < 0.05, statistical differences between males and females in the strain.

**Table 2:** Mean values (×) and standard deviation (sd) of the chemical composition of the pectoral muscles and legs of 113-week-old ducks.

Trait			Genotype (G)–Sex (S)				*p*-Values		
			Dworka (Strain D11)		Pekin (Strain P9)		G	S	G × S
			Male	Female	Male	Female			
moisture (%)	breast	× sd	71.0 ^a^ 0.1	70.4 ^a^ 0.1	71.1 ^a^ 0.1	71.8 ^b^ 0.1	<0.001	0.646	0.001
	leg	× sd	69.9 0.1	68.2 0.1	68.2 0.1	69.9 0.1	0.881	0.905	<0.001
protein (%)	breast	× sd	24.6 ^a^ 0.2	24.2 ^a^ 0.1	23.6 ^b^ 0.2	23.4 ^b^ 0.2	<0.001	0.082	<0.001
	leg	× sd	19.5 ^a^ 1.6	19.4 ^a^ 0.6	19.9 ^b^ 1.4	18.7 ^b^* 0.4	0.019	<0.001	<0.001
fat (%)	breast	× sd	2.2 0.6	2.5 0.2	2.6 0.6	2.0 0.8	0.776	0.368	0.004
	leg	× sd	5.8 1.0	7.2 * 0.7	6.2 0.6	6.8 * 0.8	0.892	0.001	0.153
collagen (%)	breast	× sd	1.4 0.1	1.4 0.1	1.4 0.1	1.5 0.1	0.148	0.809	0.111
	leg	× sd	2.1 0.3	1.8 ^a^* 0.2	2.0 0.3	1.5 ^b^* 0.1	0.026	<0.001	0.348
salt (%)	breast	× sd	0.3 ^a^ 0.1	0.2 ^a^* 0.1	0.2 ^b^ 0.1	0.1 ^b^* 0.1	0.015	0.001	0.952
	leg	× sd	1.3 ^a^ 0.2	1.1 ^a^* 0.3	0.9 ^b^ 0.4	0.9 ^b^ 0.2	<0.001	0.029	0.086

^a, b^*p* < 0.05, means with different letters are statistically different. * *p* < 0.05, statistical differences between males and females in the strain.

**Table 3:** Mean values (×) and standard deviation (sd) of selected physicochemical characteristics of the breast muscles and legs of 113-week-old ducks.

Trait			Genotype (G)–Sex (S)				*p*-Values		
			Dworka (Strain D11)		Pekin (Strain P9)		G	S	G × S
			Male	Female	Male	Female			
pH_24_	breast	× sd	6.64 0.2	6.65 0.2	6.64 0.2	6.72 0.2	0.508	0.422	0.478
	leg	× sd	6.67 0.2	6.76 0.2	6.65 0.2	6.79 0.2	0.955	0.038	0.697
conductivity -EC_24_ (mS/cm)	breast	× sd	6.7 1.3	7.0 1.4	6.1 1.7	6.5 1.7	0.234	0.409	0.977
	leg	× sd	5.3 ^a^ 2.0	6.3 ^a^* 0.9	4.3 ^b^ 1.4	5.1 ^b^* 1.4	0.015	0.042	0.748
thermal drip (%)	breast	× sd	31.4 ^a^ 2.2	30.5 ^a^ 4.0	26.5 ^b^ 4.2	28.6 ^b^ 3.7	0.014	0.468	0.179
	leg	× sd	30.7 1.2	31.9 3.2	33.9 1.7	31.9 2.1	0.118	0.689	0.101

^a, b^*p* < 0.05, means with different letters are statistically different. * *p* < 0.05, statistical differences between males and females in the strain.

**Table 4:** Mean values (×)and standard deviation (sd) of the color parameters of the breast meat and leg muscles of 113-week-old ducks.

Trait			Genotype (G)–Sex (S)				*p*-Values		
			Dworka (Strain D11)		Pekin (Strain P9)		G	S	G × S
			Male	Female	Male	Female			
color brightness (L*)	breast	× sd	36.2 3.7	35.7 2.0	35.6 4.5	36.9 4.4	0.765	0.742	0.429
	leg	× sd	37.1 4.8	38.8 4.1	36.5 4.8	35.1 4.6	0.107	0.884	0.236
red color intensity (a*)	breast	× sd	16.8 1.7	17.2 1.5	16.4 2.7	15.9 2.1	0.157	0.976	0.495
	leg	× sd	16.0 3.2	14.8 2.8	15.2 3.4	14.3 3.2	0.478	0.258	0.814
yellow color intensity (b*)	breast	× sd	4.0 ^a^ 1.8	3.4 ^a^ 1.3	2.8 ^b^ 1.3	2.0 ^b^ 1.0	0.001	0.102	0.907
	leg	× sd	6.1 2.2	6.4 2.2	6.1 3.3	6.4 2.5	0.162	0.278	0.164

^a, b^*p* < 0.05, means with different letters are statistically different. * *p* < 0.05, statistical differences between males and females in the strain.

**Table 5:** Mean values (×) and standard deviation (sd) of the microstructure elements of the greater pectoral muscle of 113-week-old ducks.

Trait		Genotype (G)–Sex (S)				*p*-Values		
		Dworka (Strain D11)		Pekin (Strain P9)		G	S	G × S
		Male	Female	Male	Female			
muscle fiber cross-sectional area (µm^2^)	× sd	267.1 33.6	239.2 * 42.0	258.9 32.5	219.5 * 30.8	0.184	0.002	0.579
muscle fiber perimeter (µm)	× sd	70.3 4.9	66.1* 5.6	68.7 3.9	63.7 * 4.3	0.141	0.001	0.792
muscle fiber horizontal (H) diameter (µm)	× sd	19.2 1.4	18.0 * 1.8	18.7 1.6	17.3 * 1.4	0.157	0.006	0.791
muscle fiber vertical (V) diameter (µm)	× sd	18.4 1.5	17.8 * 1.3	18.2 1.0	16.6 * 1.3	0.055	0.006	0.217
the ratio of the V/H diameters	× sd	0.96 0.06	0.99 0.11	0.97 0.08	0.96 0.07	0.935	0.996	0.569
*perymisium* thickness (µm)	× sd	15.6 ^a^ 1.9	17.7 ^a^ 3.6	16.2 ^a^ 2.5	12.6 ^b^ 3.0	0.007	0.367	0.001
*endomysium* thickness (µm)	× sd	1.00 ^a^ 0.05	0.96 ^a^ 0.06	0.95 ^b^ 0.07	0.90 ^b^ 0.10	0.016	0.048	0.740

^a, b^*p* < 0.05, means with different letters are statistically different. * *p* < 0.05, statistical differences between males and females in the strain.

**Table 6:** Mean values (×) and standard deviation (sd) of the texture parameters of the greater pectoral muscle of 113-week-old ducks.

Trait		Genotype (G)–Sex (S)				*p*-Values		
		Dworka (Strain D11)		Pekin (Strain P9)		G	S	G × S
		Male	Female	Male	Female			
hardness (N)	× sd	50.1 5.9	45.5 4.8	50.1 3.6	49.8 6.0	0.149	0.105	0.151
cohesiveness (-)	× sd	0.44 0.03	0.43 0.04	0.44 0.02	0.44 0.03	0.313	0.260	0.016
springiness (cm)	× sd	1.4 ^b^ 0.1	1.4 ^b^ 0.1	1.5 ^a^ 0.1	1.4 ^b^ 0.1	0.039	0.918	0.036
chewiness (N × cm)	× sd	31.5 5.4	28.3 4.8	30.1 3.0	32.0 5.8	0.383	0.597	0.094
WB shear force (N)	× sd	82.4 ^a^ 14.0	70.3 ^b^ 11.0	88.3 ^a^ 7.1	89.2 ^a^ 18.6	0.002	0155	0.097

^a, b^*p* < 0.05, means with different letters are statistically different.

## Data Availability

The data are available on request from the corresponding author.
